# Epigenetic Hierarchy within the *MAGEA1* Cancer-Germline Gene: Promoter DNA Methylation Dictates Local Histone Modifications

**DOI:** 10.1371/journal.pone.0058743

**Published:** 2013-03-05

**Authors:** Julie Cannuyer, Axelle Loriot, Grégory K. Parvizi, Charles De Smet

**Affiliations:** Group of Genetics and Epigenetics, de Duve Institute, Université Catholique de Louvain, Brussels, Belgium; Peking University Health Science Center, China

## Abstract

Gene *MAGEA1* belongs to a group of human germline-specific genes that rely on DNA methylation for repression in somatic tissues. Many of these genes, termed cancer-germline (CG) genes, become demethylated and activated in a wide variety of tumors, where they encode tumor-specific antigens. The process leading to DNA demethylation of CG genes in tumors remains unclear. Previous data suggested that histone acetylation might be involved. Here, we investigated the relative contribution of DNA methylation and histone acetylation in the epigenetic regulation of gene *MAGEA1*. We show that *MAGEA1* DNA hypomethylation in expressing melanoma cells is indeed correlated with local increases in histone H3 acetylation (H3ac). However, when *MAGEA1*-negative cells were exposed to a histone deacetylase inhibitor (TSA), we observed only short-term activation of the gene and detected no demethylation of its promoter. As a more sensitive assay, we used a cell clone harboring a methylated *MAGEA1/hph* construct, which confers resistance to hygromycin upon stable re-activation. TSA induced only transient de-repression of the transgene, and did not lead to the emergence of hygromycin-resistant cells. In striking contrast, transient depletion of DNA-methyltransferase-1 in the reporter cell clone gave rise to a hygromycin-resistant population, in which the re-activated *MAGEA1/hph* transgene displayed not only marked DNA hypomethylation, but also significant reversal of histone marks, including gains in H3ac and H3K4me2, and losses of H3K9me2. Collectively, our results indicate that DNA methylation has a dominant role in the epigenetic hierarchy governing *MAGEA1* expression.

## Introduction

DNA methylation, occurring mostly at CpG dinucleotides, is a potent mechanism of gene repression in mammalian cells [1]. Tissue-specific patterns of CpG methylation, which are established during embryo development, are generally well preserved in adult cells, but become profoundly altered in cancer cells [2]. Both gains (hypermethylation) and losses (hypomethylation) of DNA methylation can be detected within the same tumor cell. Aberrant hypermethylation in cancer often affects the promoter of tumor-suppressor genes, and has therefore been associated with loss of tumor suppressive functions [3]. DNA hypomethylation, on the other hand, has been detected on a variety of sequences within cancer genomes, including repetitive sequences [4], and was shown to contribute to the enhancement of genomic instability [5]. Surprisingly, DNA hypomethylation in tumors has been associated with transcriptional activation of only a limited number of genes, most of which have their normal site of expression restricted to the germ line [6]. This particular group of genes was termed cancer-germline (CG) genes [7]. In human, CG genes comprise about 50 genes or gene families exerting a variety of cellular functions [8]. Activation of these genes has been reported in a wide range of tumor types, including lung cancer, head and neck cancer, bladder cancer, and melanoma. One important consequence of the activation of CG genes in tumors is the production of tumor-specific antigens, which can be recognized by cytolytic T lymphocytes [9]. Clinical trials of anti-cancer vaccination targeted against such antigens are underway [10]. Conceivably, understanding the mechanisms that contribute to CG gene regulation may facilitate development of gene-induction strategies, which would render tumor cells more vulnerable to immunotherapy.

The process leading to DNA hypomethylation and subsequent activation of CG genes in tumors remains unclear [11]. One possibility is that DNA demethylation at CG genes is a consequence of alterations at the level of histone proteins, the core components of chromatin. Specific residues within histone tails undergo a variety of chemical modifications, including acetylation and methylation, which have an impact on chromatin structure and transcription [12]. Several histone modifications also appear to regulate DNA methylation states [13]. Repressive histone marks, such as methylation of histone H3 lysine 9 or 27 (H3K9 or H3K27), were shown to dictate deposition of DNA methylation at specific loci, by favoring local recruitment of DNA methyltransferases [14], [15]. On the other hand, activating histone modifications such as histone acetylation or methylation of histone H3 lysine 4 (H3K4) appear to exclude the DNA methylation machinery [16], [17]. Hence, it was tempting to propose that DNA demethylation at CG genes might be a consequence of alterations at the level of histone modifications.

Previous studies revealed that CG gene promoters are often associated with H3K9 and H3K27 methylation in non-expressing cells. These repressive modifications are generally lost upon activation of CG genes in tumor cells, and replaced by active histone marks, such as histone acetylation and H3K4 methylation [18], [19]. A crucial question is whether changes at the level of histone modifications are a cause or a consequence of CG gene promoter DNA demethylation in tumor cells. Studies using inhibitors of H3K9 and H3K27 methyltransferases showed that these were on their own unable to induce significant DNA demethylation and activation of CG genes [18], [19]. Similar results were obtained following inhibition of H3K4 demethylases [19]. In contrast, several studies reported that CG gene expression could be induced in cells treated with an inhibitor of histone deacetylases [20]–[22]. In one report [20], but not in the others, was the treatment shown to induce DNA demethylation of a CG gene promoter. These observations raised therefore the possibility that gains in histone acetylation might be a sufficient trigger to induce DNA demethylation and activation of CG genes in tumor cells.

In the present study, we evaluated the potential of histone acetylation to induce DNA demethylation and activation of gene *MAGEA1*, a well-characterized member of the CG group of genes. This was assessed by testing the effect of trichostatin A (TSA), a histone deacetylase inhibitor, in non-expressing melanoma cells and in a cell clone harboring a methylated *MAGEA1/hph* construct, which allows selection (hygromycin resistance) of cells where activation of the transgene occurred. In addition, this sensitive reporter cell system was used in a reverse experiment, in which we evaluated the ability of an inhibitor of DNA methylation to induce changes of histone marks within the *MAGEA1* promoter.

## Results

### Histone changes associated with *MAGEA1* activation in melanoma cell lines

**Figure 1:**
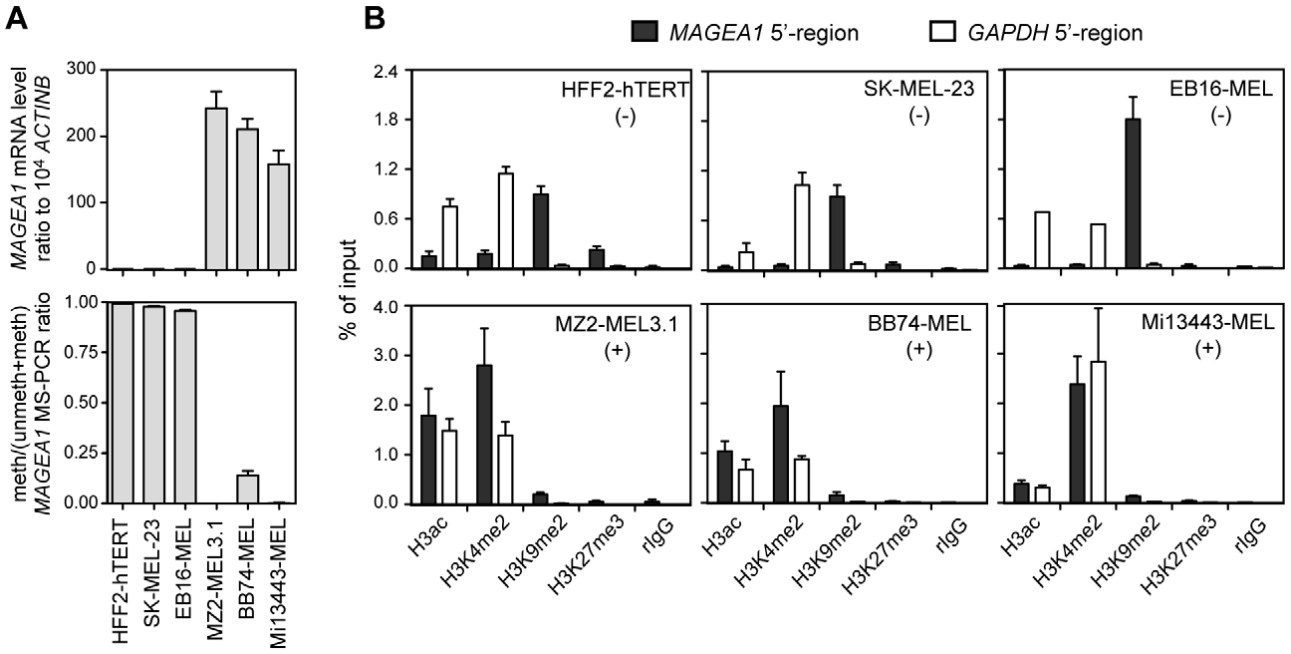
Histone changes associated with the activation of gene *MAGEA1* in melanoma cell lines. *A*, *MAGEA1* mRNA expression levels (upper panel) and 5′-region DNA methylation levels (lower panel) were evaluated in three non-expressing cell lines (HFF2-hTERT, SK-MEL-23 and EB16-MEL) as well as in three expressing cell lines (MZ2-MEL3.1, BB74-MEL and Mi13443-MEL). Values represent the mean (± sem) of two independent qRT-PCR or qMS-PCR experiments, each in duplicate. *B*, ChIP-quantitative PCR was applied to the same cell lines to evaluate enrichment of the indicated histone modifications within either the *MAGEA1* 5′-region or the *GAPDH* promoter (representing a ubiquitously active promoter). Data derive from at least two independent ChIP experiments, with two duplicate qPCR measures in each case.

In order to identify histone modification changes associated with *MAGEA1* activation, we conducted chromatin immunoprecipitation (ChIP) experiments in non-expressing and expressing cell lines. Experiments were performed on immortalized human foreskin fibroblasts (HFF2-hTERT) and two melanoma cell lines that do not express *MAGEA1* (SK-MEL-23 and EB16-MEL), as well as on three melanoma cell lines that do express *MAGEA1* (MZ2-MEL3.1, BB74-MEL and Mi13443-MEL). As expected, evaluation of *MAGEA1* promoter DNA methylation levels by quantitative MS-PCR in these cell lines, demonstrated high methylation levels in non-expressing cells, and low methylation in expressing cells (Fig. 1A). ChIP experiments, examining the *MAGEA1* 5′-region, revealed preferential enrichment of dimethylated H3K9 (H3K9me2) in *MAGEA1*-negative versus *MAGEA1*-positive cell lines (Fig. 1B). In striking contrast, acetylation of histone H3 (H3ac) and dimethylation of H3K4 (H3K4me2) within the *MAGEA1* 5′-region showed a marked increase in the three expressing cell lines, as compared to the non-expressing cells (Fig. 1B). Significant enrichment of trimethylated H3K27 (H3K27me3) within the *MAGEA1* 5′-region was observed in HFF2-hTERT cells, but not in any of the other cell lines. Together, our results suggested that DNA demethylation and activation of *MAGEA1* in melanoma cells is associated with loss of H3K9me2, and gains of H3ac and H3K4me2 within the 5′-region of the gene. This was consistent with a potential role of histone acetylation in the epigenetic activation of *MAGEA1* in tumor cells.

### TSA induces transient activation of *MAGEA1* in melanoma cells

**Figure 2:**
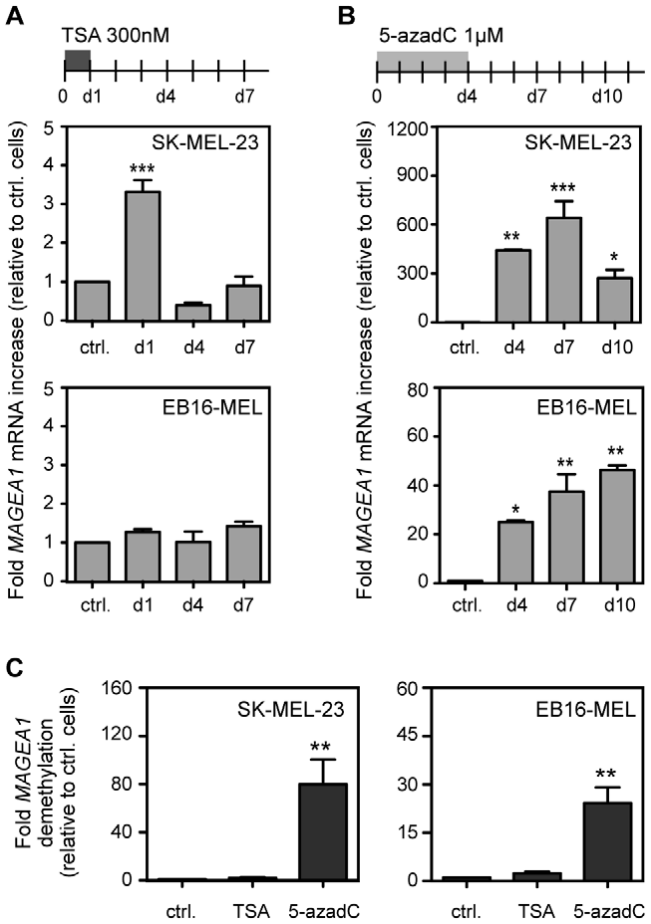
TSA induces transient activation of *MAGEA1* in melanoma cells. SK-MEL-23 and EB16-MEL cell lines were exposed to either 300 nM TSA during 24h (*A*), or 1 µM 5-azadC during 96h (*B*), and RNA was extracted from the cells at days 1, 4 and 7 after TSA treatment or at days 4, 7, 10 after 5-azadC treatment. *MAGEA1* expression levels were determined by quantitative RT-PCR. Values, which derive from three independent experiments, were normalized by the *ACTINB* expression level, and are expressed relative to the levels found in non-treated cells (ctrl). * *P*<0.05, ** *P*<0.01, *** *P*<0.001. *C*, The effect of TSA and 5-azadC on *MAGEA1* 5′-region DNA demethylation was assessed by applying MS-PCR to DNA samples extracted 10 days after the beginning of the treatments. Data (fold demethylation) correspond to the relative amount of unmethylated sequences in treated cells reported to that in untreated cells (ctrl). Values represent the mean (± sem) of three independent qMS-PCR experiments. * *P*<0.05, ** *P*<0.01.

In order to evaluate the contribution of histone acetylation in the epigenetic regulation of *MAGEA1*, we exposed SK-MEL-23 and EB16-MEL cell lines to the histone deacetylase (HDAC) inhibitor TSA, and examined its effect on the transcription and DNA methylation levels of the gene. In SK-MEL-23 cells, exposure to TSA was associated with a 3.3 fold increase in *MAGEA1* mRNA level, when assessed one day after the treatment. However, this effect was lost at days 4 and 7 after treatment (Fig. 2A). In EB16-MEL, we were unable to detect any significant activation of *MAGEA1* after TSA (Fig. 2A), which was consistent with previous data showing that the effect of TSA on *MAGEA1* activation is cell-type dependent [22]. For comparison, both cell lines were treated with the DNA methylation inhibitor 5-aza-2′-deoxycytidine (5-azadC). Because this drug induces replication-dependent DNA demethylation, the treatment was maintained during four days before analysis. Quantitative RT-PCR experiments revealed that 5-azadC (1 µM) induced significant *MAGEA1* expression in both cell lines. In SK-MEL-23 cells, the level of *MAGEA1* mRNA observed after 4 days of 5-azadC treatment was 135-fold higher than that observed after one day of TSA treatment. Moreover, in both cell lines, the 5-azadC-induced activation of *MAGEA1* was still detected at days 3 and 6 following removal of the drug (Fig. 2B).

Consistent with our expression studies, quantitative MS-PCR results revealed that TSA induced no significant decrease in the *MAGEA1* promoter methylation level, in either SK-MEL-23 or EB16-MEL cell lines (Fig. 2C). Significant demethylation of the *MAGEA1* promoter was instead observed in both cell lines after 5-azadC treatment (Fig. 2C).

Altogether, these results suggested that whereas TSA can induce transient de-repression of *MAGEA1* in initially non-expressing cells, it does not lead to DNA demethylation and long-term transcriptional activation of the gene.

### Histone modifications associated with an *in vitro* methylated *MAGEA1* transgene

Our inability to detect TSA-induced demethylation and long-term activation of gene *MAGEA1*, as reported here above, might be due to experimental limitations. It is possible, indeed, that the epigenetic impact of TSA on *MAGEA1* occurred in only a small proportion of the cells, making it very difficult to detect significant gene activation and DNA methylation changes in the treated cell population. Moreover, overt epigenetic activation of *MAGEA1* by TSA might require the presence of appropriate transcription factors, which may not be present in SK-MEL-23 or EB16-MEL cell lines. We have shown, indeed, that long-term activation of *MAGEA1* requires not only a DNA demethylation process, but also the presence of transcriptional activators to prevent subsequent remethylation of the promoter [23].

**Figure 3:**
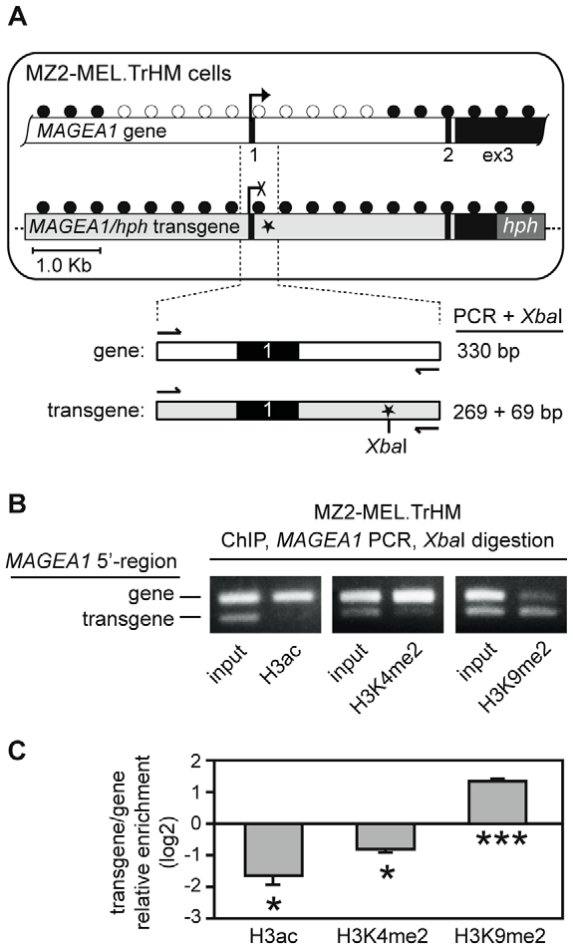
Histone modifications associated with an *in vitro* methylated *MAGEA1* transgene. *A*, Schematic representation of the structure and DNA methylation status of the active unmethylated (empty circles) *MAGEA1* gene and the inactive methylated (filled circles) *MAGEA1/hph* transgene in MZ2-MEL.TrHM cells. Black boxes correspond to the *MAGEA1* exons, the dark gray box within the *MAGEA1/hph* transgene represents the *hph* transcription unit, and the asterisk (*) is the site where a 12-bp tag sequence (carrying a *Xba*I restriction site) was inserted. The lower panel is an enlargement of the amplicon that was amplified in ChIP experiments, and indicates the expected fragment sizes following *Xba*I digestion. *B*, ChIP experiments were applied to MZ2-MEL.TrHM. The resulting *MAGEA1* amplicons were digested with *Xba*I and separated on agarose gels, thereby revealing relative enrichment of the indicated histone modifications within either the *MAGEA1* gene (upper band) or the *MAGEA1/hph* transgene (lower band). *C*, Relative enrichment of histone marks on the transgene was deduced by quantifying band intensities in gel electrophoresis pictures (ImageJ software), and calculating the lower/upper ratio. Data, which were normalized by the lower/upper ratio in input samples, derive from at least two independent ChIP experiments, with two PCR/XbaI/electrophoresis analyses in each case. * *P*<0.05, *** *P*<0.001.

We therefore decided to re-assess the effect of TSA on a previously established cell system (MZ2-MEL.TrHM) containing a selectable *MAGEA1* construct [24]. MZ2-MEL.TrHM cells contain a transgene (*MAGEA1/hph*) comprising a large portion of the *MAGEA1* locus (including the promoter region) followed by the sequence encoding resistance to hygromycin (*hph*; Fig. 3A). The transgene was methylated *in vitro* before transfection, and remains methylated and silent in MZ2-MEL.TrHM cells, which are therefore sensitive to hygromycin. However, we showed that hygromycin-resistant (hygro^r^) cell clones emerge when the *MAGEA1/hph* transgene becomes stably activated, for instance following transient treatment with 5-azadC [24]. Importantly, MZ2-MEL.TrHM cells were derived from a *MAGEA1*-expressing melanoma cell line. These cells therefore contain all necessary transcription factors to ensure long-term activation of the gene promoter, as evidenced by the presence of an active and unmethylated endogenous *MAGEA1* gene.

We first conducted ChIP experiments to identify histone modifications associated with the silent *MAGEA1/hph* transgene in MZ2-MEL.TrHM cells. The presence of a tag sequence in the *MAGEA1/hph* transgene, inserted at position +158 relative to the transcription start site and containing a *Xba*-I restriction site, allowed us to distinguish *MAGEA1* amplicons originating from either the exogenous transgene or the endogenous gene. Thus, MZ2-MEL.TrHM chromatin-derived *MAGEA1* amplicons were digested with *Xba*-I, thereby yielding an upper band (330 bp) deriving from the endogenous *MAGEA1* gene and a lower band (269 bp) deriving from the *MAGEA1/hph* transgene following electrophoresis in agarose (Fig. 3A, B). By applying this procedure to the analysis of ChIP-derived chromatin samples, we found that H3ac and H3K4me2 histone marks were strongly depleted within the *MAGEA1/hph* transgene, as compared with the endogenous *MAGEA1* gene. In contrast, the repressive H3K9me2 mark appeared predominantly enriched within the transgene (Fig. 3B, C). These results indicate that, following integration into the MZ2-MEL.TrHM genome, the *in vitro* methylated *MAGEA1/hph* transgene adopted histone marks typically associated with the repressed state of the *MAGEA1* gene in non-expressing cells.

### Lack of long-term activation of *MAGEA1/hph* following TSA treatment

**Figure 4:**
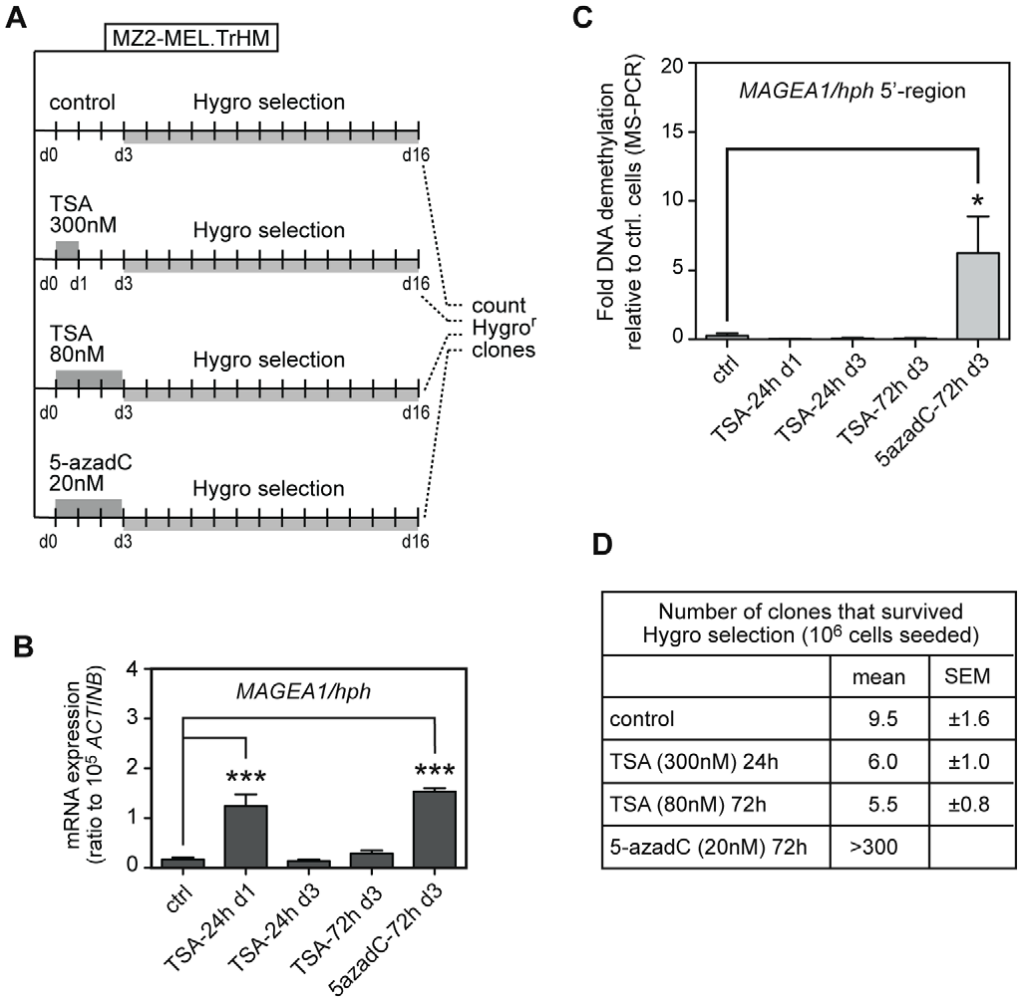
Lack of long-term activation of *MAGEA1/hph* following TSA treatment. *A*, Schematic outline of the experiment. MZ2-MEL.TrHM cells were treated or not (control) with 300 nM of TSA for 24h, 80 nM of TSA for 72h, or with 20 nM of 5-azadC for 72h. After three days, 10^6^ cells from each group were transferred into two flasks (75 cm^2^) and were selected in a medium containing hygromycin (180 µg/mL) during 13 days. *B*, The level of expression of the *MAGEA1/hph* transgene was quantified by qRT-PCR at the indicated time point (d1 or d3) in each group of cells. Data represent the mean (± sem) of al least three independent experiments. *** *P*<0.001. *C*, The level of DNA demethylation of the *MAGEA1/hph* 5′-region in the different groups of cells was evaluated by quantitative MS-PCR, using primers that specifically amplify the tagged transgene sequence. The data (fold DNA demethylation) were calculated as in Fig 2C, and correspond to the mean (± sem) of at least three independent experiments. * *P*<0.05. *D*, The number of clones that survived hygromycin selection were counted at day 16 in the three groups of cells. Data derive from at least two independent experiments, each in duplicate.

As we found low histone acetylation within the silent *MAGEA1/hph* transgene, we tested the ability of TSA to induce stable activation of the transgene, and hence the emergence of hygro^r^ clones among the treated MZ2-MEL.TrHM cell population. In addition to the conventional 24h TSA treatment, a 72h-long TSA exposure was applied to MZ2-MEL.TrHM cells, as it was reported that prolonged treatment with HDAC inhibitors may in some cases be necessary to induce localized DNA demethylation [25]. The concentration of TSA in the 72h treatment was reduced to 80 nM, because higher doses were found to kill most cells during this time period. Besides TSA-treated cells, we also analyzed cells treated with a suboptimal dose of 5-azadC (20 nM; Fig. 4A). 5-azadC at this dose was found to induce *MAGEA1/hph* mRNA expression at a level comparable to that observed at day 1 following the 24h TSA treatment (Fig. 4B). Therefore, treatment with the suboptimal dose of 5-azadC allowed us to verify if our hygromycin selection procedure was still effective in conditions where only low-level activation of the transgene occurred.

Quantitative RT-PCR experiments revealed that whereas low but significant *MAGEA1/hph* mRNA induction was observed at day 1 following the 24h TSA treatment, this induction was already lost two days later (Fig. 4B). MZ2-MEL.TrHM cells that had been exposed to the 72h TSA treatment displayed a very modest increase in *MAGEA1/hph* mRNA expression (not significant, *p = 0.1428*), as compared with control cells (Fig. 4B). This low level of induction was likely attributable to the reduced concentration of TSA in this condition. Moreover, quantitative MS-PCR directed specifically towards the transgenic *MAGEA1* 5′-region, revealed no demethylation of this DNA sequence in either of the TSA-treated cell populations, as compared with untreated cells (Fig. 4C). In contrast, cells treated with the suboptimal dose of 5-azadC displayed moderate, albeit significant, DNA demethylation of the *MAGEA1/hph* transgene.

In order to detect rare cells where *MAGEA1/hph* transgene activation might have occurred, and which may have remained invisible in our RT-PCR and MS-PCR analyses, we submitted the differently treated MZ2-MEL.TrHM cell populations to selection in hygromycin. After 13 days of selection, hygro^r^ colonies were counted. The mean frequency of hygro^r^ clones obtained following either the 24h or the 72h TSA treatment (6×10^-6^ and 5.5×10^-6^, respectively) was not higher than that observed for untreated control cells (9.5×10^-6^), and corresponded therefore to the background level of spontaneously hygro^r^ revertant cells (Fig. 4D). In comparison, treatment with the suboptimal dose of 5-azadC resulted in the emergence of a much higher number of hygro^r^ clones (>3×10^-4^). Taken together, these results indicate that TSA does not induce DNA demethylation and stable activation of the *MAGEA1/hph* transgene, not even in a small proportion of the treated MZ2-MEL.TrHM cells.

### DNA demethylation induces reversal of histone marks in *MAGEA1/hph*

**Figure 5:**
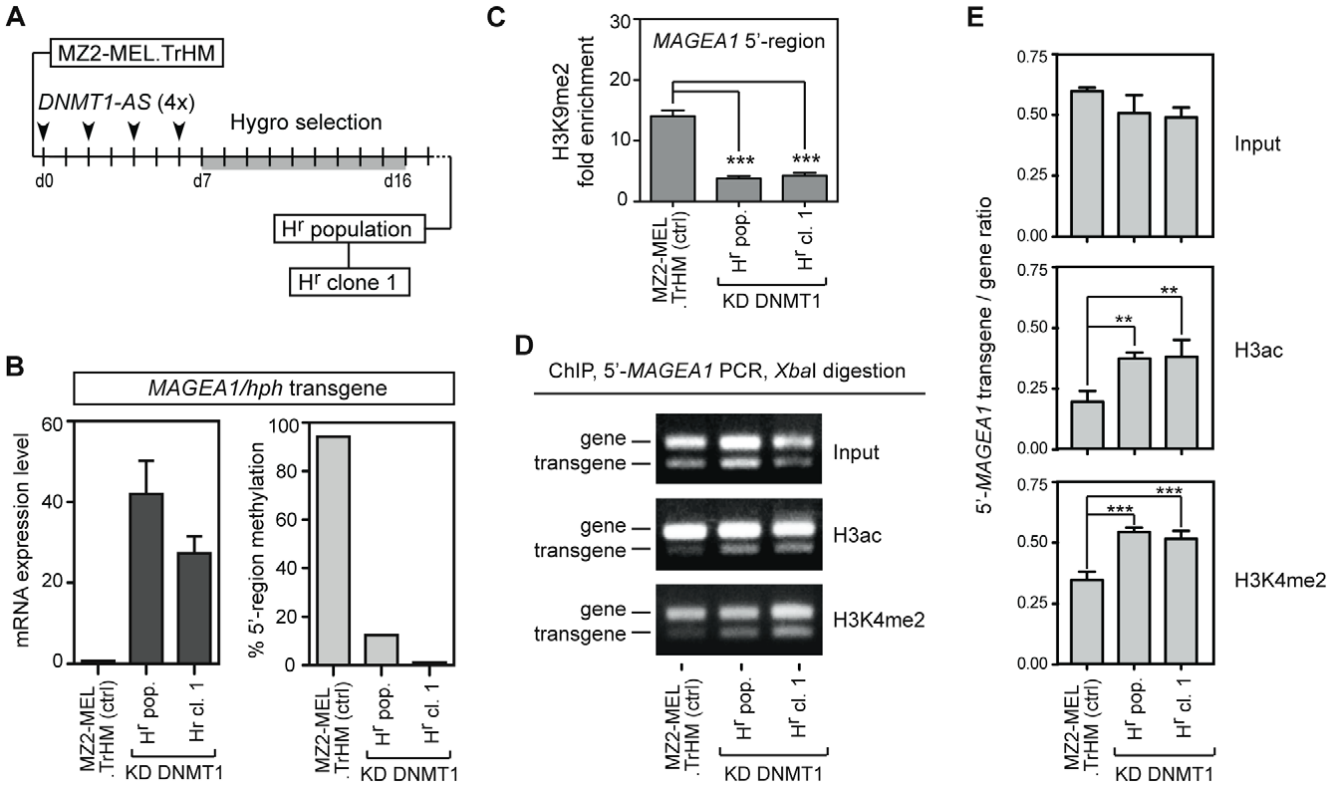
DNA demethylation induces reversal of histone marks in *MAGEA1/hph*. *A*, Schematic outline of the derivation of the H^r^ population and H^r^ clone 1 from MZ2-MEL.TrHM cells (see reference [24] for details). MZ2-MEL.TrHM cells were repeatedly tranfected with antisense oligonucleotides directed against *DNMT1* (*DNMT1-AS*) during 7 days, and were thereafter transferred into medium containing hygromycin. After 9 days of hygromycin selection, a resistant population emerged (H^r^ population), and a clone (H^r^ clone 1) was isolated from this population by limiting dilution. The H^r^ population and H^r^ clone 1 were subsequently cultured without hygromycin selection. *B*, The mRNA expression level (ratio to 10^4^ *ACTINB*) and 5′-region DNA methylation status (% methylated CpGs) of the *MAGEA1/hph* transgene were determined in MZ2-MEL.TrHM cells, the H^r^ population and H^r^ clone 1 by qRT-PCR and bisulfite sequencing, respectively. *C*, ChIP-qPCR was used to evaluate enrichment of H3K9me2 within the *MAGEA1/hph* transgene in the three groups of cells (see text for details). Fold enrichment levels were obtained by reporting the *MAGEA1* 5′-region enrichment values to that of the *GAPDH* 5′-region in the same sample. Data represent the mean (± sem) of two to three ChIP experiments, with two duplicate qPCR measurements in each case. *** *P*<0.001. *D*, The ChIP/PCR/*Xba*I procedure (see Fig. 3A, B) was applied to evaluate enrichment of H3ac and H3K4me2 within the 5′-region of the *MAGEA1/hph* transgene in the three group of cells. *E*, ImageJ analyses of gel electrophoresis pictures were used to quantify the *MAGEA1* 5′-region transgene/gene ratio. Data represent the mean (± sem) of two independent ChIP experiments, with two PCR/XbaI/electrophoresis analyses in each case. ** *P*<0.01, *** *P*<0.001.

Considering previous studies by others [18], [19], and the observations we described here above, it appears unlikely that changes at the level of histone modifications can on their own be a sufficient trigger to cause DNA demethylation and long-term activation of the *MAGEA1* gene. It was therefore reasonable to propose that the reversal of histone marks associated with *MAGEA1* activation is instead a consequence of DNA demethylation. To test this hypothesis, we resorted to a previously established hygro^r^ sub-population of MZ2-MEL.TrHM cells (H^r^ population, Fig. 5A and [24]), which had been obtained following transient exposure of the cells to antisense oligonucleotides directed against *DNMT1*, the predominant DNA methyltransferase in these cells [24]. We decided to conduct ChIP experiments on the H^r^ population in order to determine the impact of DNA demethylation on H3ac, H3K4me2 and H3K9me2 histone marks within the *MAGEA1/hph* transgene. Analyses were also performed on a cell clone (H^r^ clone 1), which had been isolated from the H^r^ population (Fig. 5A). Quantitative RT-PCR and sodium bisulfite sequencing confirmed robust transcriptional activation and DNA demethylation of the *MAGEA1/hph* transgene in the H^r^ population and H^r^ clone 1 (Fig. 5B).

For the analysis of H3K9me2, ChIP samples were submitted to quantitative PCR with primers not distinguishing between endogenous and transgenic *MAGEA1* sequences. Lack of H3K9me2 within the endogenous active *MAGEA1* gene in MZ2-MEL.TrHM cells (see Fig. 3B, C) implied, however, that most ChIP-derived amplicons would originate from the *MAGEA1/hph* transgene. Results, which are depicted in figure 5C, were consistent with a decreased enrichment of H3K9me2 within the 5′ region of *MAGEA1/hph* in the H^r^ population and in the H^r^ clone 1, as compared with control MZ2-MEL.TrHM cells. For the analysis of H3ac and H3K4me2, we resorted to the ChIP-PCR-*Xba*I procedure described above (Fig. 3A). The results showed that whereas the *MAGEA/hph* transgene was not associated with H3ac and H3K4me2 in MZ2-MEL.TrHM control cells, it displayed significant enrichment of these two activation marks in the H^r^ population and in the H^r^ clone 1 (Fig. 5D, E). Altogether, these results indicate that transient depletion of DNMT1 in MZ2-MEL.TrHM cells, resulted in the emergence of hygro^r^ cells, in which the re-activated *MAGEA1/hph* locus displayed not only marked DNA hypomethylation, but also significant reversal of its histone modification profile towards an active configuration.

## Discussion

Genome hypomethylation is frequently observed in tumor cells, and has been associated with malignant progression. Yet, the mechanisms underlying this epigenetic alteration are still unknown. Considering the tight link existing between DNA methylation and histone modifications, it was reasonable to propose that DNA hypomethylation in tumors might be a consequence of changes occurring at the level of histone marks. Alterations of histone modification profiles have indeed been observed in different tumor types, and have in some cases been associated with mutations in histone modifying enzymes [26].

In the present study, we analyzed the relationship between DNA methylation and histone modifications within the *MAGEA1* gene, as it belongs to the unique group of CG genes, for which promoter hypomethylation was frequently observed in a variety of tumors and was consistently associated with transcriptional activation [11]. It has been previously reported that demethylation and activation of CG genes in tumors is generally associated with losses of repressive histone marks, and gains in activating histone marks [18], [19]. Such association was confirmed in our present study, as we found that *MAGEA1* demethylation and activation in melanoma cells correlated with losses of H3K9me2, and gains in H3ac and H3K4me2. Previous studies however, showed that the expression of *MAGEA1* could not be activated by simply modulating H3K9me2 or H3K4me2 levels, suggesting that these two histone modifications play only a secondary role in the epigenetic regulation of this gene [18], [19]. Inhibitors of histone deacetylases, including TSA, were instead found to induce activation of *MAGEA1*, implying that histone acetylation might contribute more actively to the epigenetic regulation of this gene [22], [27]. Our present data however, indicate that TSA treatment leads to only transient activation of *MAGEA1* and does not induce DNA demethylation within the gene promoter.

Our study provides an in-depth analysis of the effect of TSA on *MAGEA1* activation, since we tested its impact not only in non-expressing cell lines, but also in the MZ2-MEL.TrHM cell clone, which contains an *in vitro* methylated transgene comprising the 5′ portion of *MAGEA1* followed by the sequence encoding resistance to hygromycin. This cell system provides high sensitivity, as it permits selection of cells (even if they are rare), in which activation of the *MAGEA1* promoter occurred. Additionally, because the cells express the endogenous *MAGEA1* gene, they obviously contain all necessary factors to ensure transcriptional activation of the transgenic *MAGEA1* promoter, once it is unleashed from chromatin constraints. Importantly, we showed that, unlike the endogenous *MAGEA1* gene promoter, the exogenous *MAGEA1* transgene promoter in MZ2-MEL.TrHM cells was associated with high levels of H3K9me2 and low levels of H3ac and H3K4me2. This suggests that, upon its integration into the host chromatin, the *in vitro* methylated *MAGEA1* transgene adopted the repressive histone modification profile typically associated with the *MAGEA1* gene in constitutively non-expressing cells. Following exposure of MZ2-MEL.TrHM cells to TSA, we observed only short-term activation of the *MAGEA1* transgene promoter, and detected no induction of hygromycin-resistant cell clones. In addition, we found no evidence of DNA demethylation within the transgene. Together, our data suggest that HDACs act as downstream effectors of DNA methylation at the *MAGEA1* promoter. A plausible scenario is that the presence of HDACs in this region depends on their association with methylated-CpG-binding proteins, including MBD1 and MeCP2, which remain in place as long as the CpG sites are methylated [28]. Therefore, HDAC activities would be rapidly restored within the *MAGEA1* promoter after removal of TSA.

Remarkably, we observed that specific depletion of DNMT1 in MZ2-MEL.TrHM cells led not only to DNA demethylation within the *MAGEA1* transgene, but also to local reversal of the repressive histone modification profile towards an active configuration. This important observation strongly supports the notion that DNA methylation dictates local histone modifications within the *MAGEA1* promoter region, and is therefore a dominant component of the epigenetic regulation of the gene. Of note, epigenetic reversal within the transgenic *MAGEA1* promoter was observed in the absence of continuous hygromycin selection, indicating that it does not require a selective pressure to be maintained subsequently to the transient phase of DNMT1 depletion. This contrasts with a previous study, which used a transgenic cassette based on the erythroid-specific *ß^A^-globin* promoter, and where continuous hygromycin selection was required to maintain the promoter unmethylated and associated with active histone marks [29].

While connections between histone modifications and DNA methylation have been well documented during the past years, it still remains unclear how these different epigenetic layers influence each other in deposition [30]. The classical view is that, upon gene silencing, histone modifications constitute a first layer of repression, which dictates the subsequent deposition of DNA methylation. DNA methylation is therefore believed to serve merely as an extra lock-off mechanism for already silenced genes [1]. This setting is clearly illustrated by the mode of repression of the *Oct-3/4* gene in differentiating mouse embryonic stem cells, which was shown to require histone deacetylation and H3K9 methylation prior to DNA methylation [31]. Consistently, mutant cells lacking DNA methylation still displayed *Oct-3/4* repression and local H3K9 methylation [31]. Another illustration of a predominant role of histone modifications was provided by a report demonstrating that HDAC inhibitors were sufficient to induce DNA demethylation and re-activation of genes that were silenced by promoter hypermethylation in tumor cell lines [25]. Collectively, the available data suggest that DNA methylation acts as an accessory mechanism of epigenetic regulation in most cases [32], [33]. However, it remains possible that DNA methylation plays a more dominant role at certain genomic loci [30]. Together with previous work by others [18], [19], our present study provides strong evidence that the *MAGEA1* gene promoter represents a genomic locus where DNA methylation exerts such a dominant epigenetic function. This particular condition of *MAGEA1* may explain why it belongs to the few genes that frequently become activated in tumors displaying DNA hypomethylation. It is predicted indeed that for *MAGEA1*, and probably also for other CG genes, DNA demethylation can be a sufficient trigger to induce complete epigenetic reversal and long-term transcriptional activation. This would not be the case for other genes where DNA methylation plays only a secondary role, as these genes would retain repressive histone marks even when they become demethylated.

Understanding the mechanisms that lead to DNA hypomethylation and CG gene activation in tumors is important in the field of cancer therapy. Indeed, antigens encoded by CG genes are ideal targets for cancer immunotherapy because of their lack of expression in normal somatic tissue and their widespread expression in human tumors. Currently, vaccines directed against such antigens are in clinical trials. These vaccines have been reported to induce immune responses, accompanied by clinical benefit in some patients [10]. An interesting possibility to augment vaccination efficiencies would be to use epigenetic modulators that increase the number of expressed CG genes, thereby rendering tumor cells more vulnerable to the immune system. To this end, DNA methylation inhibitors as well as HDAC inhibitors are being considered [34]. Our data suggest however that HDAC inhibitors have only a short-term effect on CG gene expression. Such transient effect of TSA was also observed when used in combination with a DNA methylation inhibitor (data not shown). This narrow window of activation should be taken into account when considering an anti-cancer treatment schedule that combines tumor antigen vaccines and epigenetic drugs, including HDAC inhibitors.

## Materials And Methods

### Cell lines

Human melanoma cell lines MZ2-MEL3.1, BB74-MEL and EB16-MEL, SK-MEL-23, and Mi13443 were obtained from the Brussels Branch of the Ludwig Institute for Cancer Research. They were derived and cultured as previously described [35], [36]. HFF2 human foreskin fibroblasts (from ATCC, ref. SCRC-1042) that had been transduced with hTERT (HFF2-hTERT) were received from Dr. A. Decottignies (De Duve Institute, Brussels, Belgium) and were cultured as previously described [37]. Cell cultures were maintained at 37°C in a humidified atmosphere of 5% CO_2_ for HFF2-hTERT cells or 8% CO_2_ for the other cells.

### 5-azadC and TSA treatments of cell lines

For 5-aza-2′-deoxycytidine (5-azadC) treatments, cells were seeded (10^6^ per 75 cm^2^ flask) in medium containing 20 nM or 1 µM of 5-azadC (Sigma-Aldrich Chemie GmbH, Steinheim, Germany) and the DNA and RNA were extracted from the cells after three or four days of treatment respectively. For trichostatin A (TSA) treatments, cells were seeded (10^6^ per 75 cm^2^ flask) in medium containing 300 nM or 80 nM of TSA (Cayman Chemical, Ann Arbor, MI) and the DNA and RNA were extracted from the cells after 24 or 72 hours of treatment, respectively. RNA was extracted using the TriPure Isolation Reagent protocol (Roche Diagnostics GmbH, Manheim, Germany) and finally resuspended in 20 µL of RNase-Free water. DNA was extracted in a SDS-Proteinase K lysis buffer, as previously described [38].

### Hygromycin selection in MZ2-MEL.TrHM cells

Construction of MZ2-MEL.TrHM cell clone has been previously described [24]. Long-term activation of the *MAGEA1/hph* transgene in these cells after TSA or 5-azadC treatment was evaluated by counting the number hygromycin-resistant cells. To this end, treated cells were transferred in a medium containing 180 µg/mL of Hygromycin B (InvivoGen, San Diego, CA) and after 13 days, the number of clones that survived hygromycin selection were counted. Derivation of the H^r^ population and H^r^ clone 1 from MZ2-MEL.TrHM cells that had been exposed to anti-DNMT1 antisense oligonucleotides has been previously described [24].

### Quantitative RT-PCR

Reverse transcription was performed on 2 µg of total RNA using oligo(dT) primers as described elsewhere [38]. Quantitative RT-PCR amplifications were performed using the qPCR Core kit reaction mix, according to the manufacturer’s instructions (Eurogentec, Seraing, Belgium). The primers and specific 5′-FAM/3′-TAMRA labeled probes were synthesized commercially (Eurogentec). Those used for the amplification of the endogenous *MAGEA1* and the *ACTINB* genes were described elsewhere [38]. For the *MAGEA1/hph* transgene, we used the following primers and probe : 5′-CCAACCCAGAGGACAGGATT (sense ; exon 2), 5′-GCCGATAAACATAACGATCTT (antisense ; *hph* sequence), 5′-6FAM-CTCCTATGTCCTTGTCACCTGCCTA-TAMRA-3′ (probe). All samples were analyzed in duplicates. Expression levels were normalized to that of *ACTINB*.

### Bisulfite Genomic Sequencing and quantitative MS-PCR

For the analysis of the methylation level of the endogenous *MAGEA1* gene, we resorted to quantitative methylation-specific PCR (qMS-PCR). The reaction conditions and validation of the *MAGEA1* qMS-PCR, which involved nested PCR, have been described elsewhere [39]. Methylation analyses of the *MAGEA1/hph* transgene were performed either by bisulfite sequencing as described previously [24], or by qMS-PCR. Conditions for qMS-PCR of the *MAGEA1/hph* transgene were similar to those for the endogenous *MAGEA1* gene, except that the antisense primer in the first PCR step was replaced by a primer that specifically recognizes a tag sequence inserted within the transgenic *MAGEA1* sequence (5′-GAGAYGTTTTTTYGYGTTTTAGA). To evaluate the relative levels of demethylation of either *MAGEA1* or *MAGEA1/hph* following TSA or 5-azadC treatment, we first calculated the ratio of unmethylated / (methylated + unmethylated) inferred from the -∂CTs in each sample, and then reported these ratios to that in the control cells.

### ChIP assays and antibodies

Chromatin immunoprecipitation (ChIP) was performed on adherent cell lines at ∼ 90% confluency in a 150 mm culture dish containing 25 mL of growth media. ChIP assays were carried out using the EZ-ChIP Kit according to the manufacturer’s instructions (Millipore, Temecula, CA) and chromatin was sheared with the Bioruptor Sonicator (Diagenode, Liège, Belgium). The chromatin was immunoprecipitated using the following antibodies : anti-acetyl-Histone H3 polyclonal antibody (06-599; Millipore), anti-dimethyl-Histone H3 (Lys 4) polyclonal antibody (39141; Active Motif, La Hulpe, Belgium), anti-dimethyl-Histone H3 (Lys 9) monoclonal antibody (Mab-154-050; Diagenode), anti-trimethyl-Histone H3 (Lys 27) polyclonal antibody (17–622; Millipore) and normal rabbit IgG (sc-2027; Santa Cruz Biotechnology Inc., Heidelberg, Germany). DNA purified from both the immunoprecipitated and pre-immune (input) samples was subjected to quantitative PCR amplification using the following primers and probes : 5′-GGCAGAGAGAAGCGAGGTT (sense primer ; -19), 5′-GGAATATTTGGGGCTCTCTA (antisense primer ; +126), and 5′-6FAM-AGGAACCTGACCCAGGCTCTGTGAG-TAMRA-3′ (probe ; -33) for the *MAGEA1* gene; and 5′-TACTAGCGGTTTTACGGGCG (sense primer ; -230), 5′-CGAACAGGAGGAGCAGAGAGCGA (antisense primer ; +46), and 5′-6FAM-AGGCCTCAAGACCTTGGGCTGGGACTG -TAMRA-3′ (probe ; -88) for the *GAPDH* gene. Results are expressed as percentage of input, which represents the ratio of immunoprecipitated DNA / input DNA inferred from the -∂CTs in each sample; or expressed as fold enrichment, which was established by calculating the ratio of % input for *MAGEA1* / % input for *GAPDH* in each sample.

After ChIP experiments in MZ2-MEL.TrHM cells, the endogenous *MAGEA1* gene as well as the *MAGEA1/hph* transgene were amplified by PCR with a common set of primers targeting the 5′-region: 5′-TCCCACCCCCACCCAGGCAGGAT (sense ; -105) and 5′-CCTGGTGTCTCTCAAGGCTTT (antisense ; +225 in MAGEA1 ; +237 in *MAGEA1/hph* due to the additional 12-bp tag sequence). ChIP-derived DNA was amplified in a 30 µL PCR reaction containing 1x DreamTaq Buffer (Fermentas GmbH, Leon-Rot, Germany), 200 µM of each dNTP (Takara, Shiga, Japan), 1% of DMSO (Merck Millipore, Darmstadt, Germany), 5 µM of each primer, and 25 units of DreamTaq Polymerase (Fermentas). *MAGEA1* amplification was performed for 36 cycles : 45 sec at 94°C, 45 sec at 66°C, 70 sec at 72°C. The PCR products were digested overnight at 37°C with 30 units of *Xba*I restriction enzyme (Fermentas) and separated on a 2% agarose gel. The relative enrichment of histone marks on the transgene was deduced by quantifying band intensities in gel electrophoresis pictures with the ImageJ software. Data are expressed as transgene/gene ratio ( =  lower/upper band ratio), and were normalized by the transgene/gene ratio in the corresponding input control.
